# Overview Article Astrocytes as Initiators of Epilepsy

**DOI:** 10.1007/s11064-022-03773-z

**Published:** 2022-10-16

**Authors:** Lukas Henning, Petr Unichenko, Peter Bedner, Christian Steinhäuser, Christian Henneberger

**Affiliations:** 1 Institute of Cellular Neurosciences, Medical Faculty, University of Bonn, 53127 Bonn, Germany; 2 German Center for Neurodegenerative Diseases (DZNE), 53127, Bonn, Germany

**Keywords:** Astrocyte, Epilepsy, Gap junction, Glutamate uptake, K^+^ spatial buffering, Adenosine kinase

## Abstract

Astrocytes play a dual role in the brain. On the one hand, they are active signaling partners of neurons and can for instance control synaptic transmission and its plasticity. On the other hand, they fulfill various homeostatic functions such as clearance of glutamate and K^+^ released from neurons. The latter is for instance important for limiting neuronal excitability. Therefore, an impairment or failure of glutamate and K^+^ clearance will lead to increased neuronal excitability, which could trigger or aggravate brain diseases such as epilepsy, in which neuronal hyperexcitability plays a role. Experimental data indicate that astrocytes could have such a causal role in epilepsy, but the role of astrocytes as initiators of epilepsy and the relevant mechanisms are under debate. In this overview, we will discuss the potential mechanisms with focus on K^+^ clearance, glutamate uptake and homoeostasis and related mechanisms, and the evidence for their causative role in epilepsy.

## Introduction

Astrocytes are a subtype of glial cell in the brain. They play many physiological roles that range from neurotransmitter uptake to the modulation of synaptic transmission and plasticity [1]. Profound changes of astrocyte properties and function in brain diseases such as epilepsy are a common and widespread finding. Such alterations of astrocytes can be found on the level of protein expression, morphology, and operation of signaling cascades, which can contribute to the phenotype and symptoms of the disease and to disease progression although many disease-specific mechanisms remain to be fully understood [2]. A key question is often whether astrocyte changes in disease are the origin of the disease or a consequence of it. For epilepsy, which is a heterogenous group of neurological disorder characterized by recurrent epileptic seizures, this is an intensely discussed question. In the following, we will therefore discuss by which mechanisms astrocytes could play a role as initiators of epilepsy and what the experimental evidence for such a causative role of astrocytes is. We will limit our discussion to temporal lobe epilepsy (TLE), a common and often drug-resistant form of epilepsy.

One strategy for revealing if astrocyte mechanisms can initiate and promote epileptic activity, is to identify astrocytic changes in epileptic tissue and then test if disruption of such candidate mechanisms affects epilepsy and if reproducing a specific astrocytic change is sufficient to induce epilepsy. Relevant information about astrocytic changes has been gained from epileptogenic brain tissue specimens surgically resected from patients with drug resistant TLE showing hippocampal sclerosis (HS). Both TLE and HS are strongly associated with an initial precipitating event such as febrile seizures, trauma, hypoxia, or brain infections [3]. In humans, chronic TLE usually does not develop immediately after such an event but following a seizure-free period that can last many years and is referred to as the latent period [4]. The latter is of particular interest for epilepsy research as during this period, pathophysiological changes occur that eventually culminate in chronic epilepsy. Since the latent period cannot be studied in human tissue, animal models are required that reproduce this typical pattern of epileptogenesis. This is also important because an astrocytic change supposedly causing the development of epilepsy needs to occur before the onset of epilepsy and epileptic neuronal activity. Also, the availability of live human tissue is limited and experimental work in live human tissue with HS is challenging. Therefore, several post-*status epilepticus* (SE) models have been investigated in which systemic or local administration of chemoconvulsants or electrical stimulation triggers SE and the development of chronic epilepsy after a latent period of days to weeks [5–8]. Accumulating evidence from these models suggests that astrocytes become dysfunctional immediately after the initial precipitating event or during the latent period. Accordingly, these changes could be causative in epileptogenesis. It is important to note here that the properties of astrocytes in acute slices from human cortical specimens (resected to gain access to the epileptogenic area) or non-sclerotic hippocampal slices from patients with ‘lesion-associated’ TLE that lack significant histopathological hippocampal alterations were remarkably similar to those from the corresponding brain areas of healthy rodents [9].

**Fig. 1:**
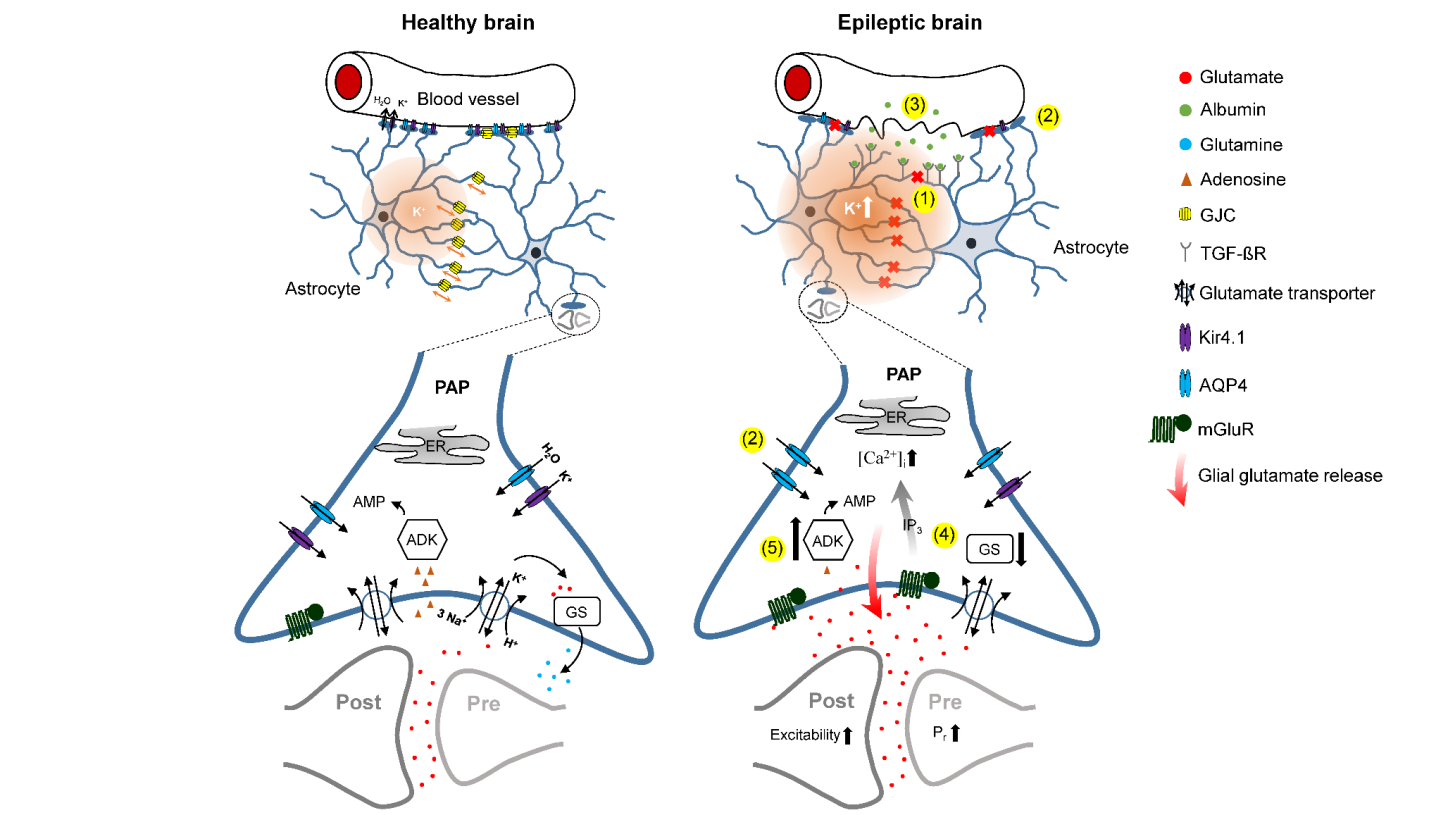
Schematic illustration of astrocyte changes in the healthy (left) vs. epileptic (right) brain. (1) In epilepsy, astrocytes lose their ability to form functional GJ-coupled networks, resulting in increased extracellular K^+^ concentrations. (2) The density of AQP4 channels along the perivascular membrane domain of astrocytes is reduced in the epileptic brain, leading to a dysregulation of water transport and a concomitant decrease in the extracellular space volume as well as an increase of the extracellular fluid osmolarity. Additionally, downregulation of astrocytic Kir4.1 channels contributes to impaired K^+^ buffering in both human and experimental epilepsy. (3) Seizure-induced disruption of the BBB results in albumin extravasation and subsequent TGF-βR-mediated astrocyte dysfunction. (4) Perturbations in astrocyte-dependent glutamate homeostasis including impaired glutamate uptake, reduced GS efficiency and aberrant glutamate release by astrocytes contribute to increased extracellular glutamate levels. (5) Overexpression of ADK in epileptic tissue decreases the ambient adenosine concentration and could amplify neuronal excitability

## Involvement of Kir4.1 Channels

There are several consistent findings regarding astrocyte changes in epileptic tissue (Fig. 1). For instance, the ability of astrocytes in human HS to fulfill one of its essential functions, the spatial buffering of K^+^ [10], has already been investigated more than 20 years ago [11–13]. These authors showed that specific inhibition of glial inwardly rectifying K^+^ (Kir4.1) channels, which mediate the passive uptake of K^+^ by astrocytes, substantially augmented stimulus induced or iontophoretically applied elevations in extracellular K^+^ in non-sclerotic human hippocampal slices but had no effect in human HS. These results provided first evidence for the disturbance of K^+^ buffering in HS. Further evidence came from comparative patch-clamp studies showing a significant reduction of astrocytic Kir currents in HS [14–16]. In line with this, significantly reduced Kir4.1 protein levels were detected by immunohistochemical and western blot analysis in patients with HS compared to non-sclerotic and autopsy controls [17–19]. Importantly, genetic linkage studies have indicated an association between missense variations in the human Kir4.1 gene (KCNJ10) and seizure susceptibility in TLE [20, 21]. For these reasons, impaired K^+^ buffering via Kir4.1 is a candidate mechanism in epileptogenesis.

Insights into the importance of Kir4.1 channels for K^+^ buffering and regulation of neuronal excitability particularly emerged from Kir4.1 knockout (KO) mice, which exhibit impaired K^+^ clearance and an epileptic phenotype [22, 23]. However, whether changes in Kir4.1 represent a causal event in epileptogenesis is still unclear because data on its expression during the latent period are inconsistent among experimental TLE models. For instance, in an albumin model downregulation of Kir4.1 transcripts and reduced K^+^ clearance were detected long before the onset of epileptic activity [24], while no changes in Kir4.1-mediated K^+^ currents could be found in hippocampal astrocytes during the latent period of experimental TLE induced by systemic injection of kainate [25].

## Uncoupling of Astrocytes

In addition to Kir4.1, spatial K^+^ buffering may also depend on the interconnection of astrocytes through gap junction channels. To our knowledge, characterization of functional gap junction coupling between human hippocampal astrocytes has been performed in only one study [6]. Here, tracer diffusion assays demonstrated a complete loss of astrocytic coupling in human HS, whereas in non-sclerotic control tissue the extent of coupling was similar to that observed in rodents [6, 9]. Interestingly, it was shown that this loss of coupling is not due to decreased expression of gap junction proteins (connexins, Cx), but rather the result of altered subcellular localization and phosphorylation of Cx43 [26]. The pathological consequences of impaired gap junctional coupling can be seen in patients with oculodentodigital dysplasia (ODDD), a rare genetic disease caused by mutations in the gene encoding Cx43. These patients present with epileptic seizures in addition to other neurological symptoms [27, 28]. These observations imply that perturbed gap junction coupling is an additional candidate mechanism in epileptogenesis. This is also suggested by a recent study revealing that gap junction coupling plays a selective role in buffering high local K^+^ increases [29], which are typical for epilepsy.

Experimental support for the importance of astrocytic gap junction channels in K^+^ clearance and neuronal hyperexcitability has been gained in hippocampal slices from transgenic mice with coupling-deficient astrocytes [30, 31] and with pharmacological disruption of gap junction communication in situ and in vivo [29, 32, 33]. Analyses of transgenic mice with coupling-deficient astrocytes further revealed that not only K^+^ buffering but also glutamate clearance is impaired when astrocyte Cxs are absent [30]. Consistently, acute slices from these mice displayed spontaneous epileptiform events and substantially increased seizure and interictal spike activity during the chronic phase of experimentally induced TLE [31, 34]. However, neither constitutive nor inducible astrocytic Cx KO mice showed spontaneous behavioral seizures or abnormal EEG activity in vivo [35, 36]. It has to be considered that in the latter studies, mice with complete or significant deletion of Cx43 and Cx30 were used, while loss of coupling in HS was not accompanied by any reduction in Cx proteins [26]. Complete loss of gap junction coupling as well as the subcellular reorganization and altered phosphorylation of Cx43 characterizing human HS could be reproduced in the intracortical kainate injection mouse model of TLE-HS [6, 26]. Intriguingly, in this model, loss of astrocyte coupling and the resulting impairment in K^+^ clearance temporally precede neuronal death and onset of spontaneous seizure activity, pointing to a causal role of astrocyte dysfunction in the initiation of TLE [6].

The signaling pathway underlying loss of astrocyte coupling in TLE remains unknown. One potential mechanism can be inferred from the observation that epilepsy is associated with a breakdown of the blood-brain barrier (BBB), which results in extravasation of serum proteins, including albumin, into the brain parenchyma [37, 38]. Extravasated albumin is endocytosed by astrocytes [39–41] via binding to transforming growth factor beta (TGFß) receptors [42–44]. Experimental albumin infusions impair interastrocytic gap junction coupling and extracellular K^+^ buffering, probably due to TGFß signaling-dependent transcriptional downregulation of astrocytic Cxs and Kir4.1 [24, 39, 43, 44]. Similarly, albumin-induced TGFß signaling has been shown to induce epileptiform activity in situ and in vivo [42, 43, 45]. In animal models of TLE, BBB opening and albumin extravasation occur within hours of the precipitating insult [46–48]. However, data examining consequences of albumin induced TGFß signaling in astrocytes in experimental TLE are limited. In a recent study performed in our lab, astrocytic albumin uptake 4 and 24 h after kainate-induced SE was negligible. Early short-term TGFβR1 kinase inhibition did not prevent seizure-induced gap junction uncoupling in astrocytes and exerted only minor effects on acute and chronic epileptiform activity [48]. In contrast, long-term treatment with the angiotensin II type 1 receptor inhibitor losartan, which also inhibits TGFβ signaling [42], reduces seizure frequency and attenuates hippocampal neurodegeneration and behavioral abnormalities in kainate-induced epilepsy in rats [49]. Together, these findings indicate that sustained inhibition of albumin-induced TGFß signaling could be necessary to affect epileptogenesis. This hypothesis is also supported by the observation that albumin extravasation is a phenomenon that persists until the chronic phase of experimental and human TLE [26]. Two other recent studies performed in rats showed that inhibition of TGFß1 signaling attenuates kainate-induced seizures and astrogliosis [50, 51]. Unfortunately, the two latter studies did not determine whether neuronal or astrocyte TGFß signaling was affected, and the outcome of TGFß inhibition on development of chronic seizure activity was also not addressed. Thus, further investigations are needed to decide whether albumin induced TGFß signaling is causally involved in astrocyte dysfunction and epileptogenesis.

## Perturbed Glutamate and Adenosine signaling

Glutamate transport and homoeostasis are also implicated in epileptogenesis and controlled by astrocytes. For instance, astrocytes are believed to mediate most of the uptake of glutamate released from neurons [52, 53], which is a central mechanism ensuring physiological excitatory neurotransmission and protection from excitotoxicity [54, 55]. Astrocytic glutamate uptake is accomplished by the glia-specific transporter EAAT1 (GLAST) and by EAAT2 (GLT-1). Glutamate is thought to be then converted into glutamine by the enzyme glutamine synthetase (GS), which is then shuttled back to neurons for the resynthesis of glutamate [53, 56]. Extracellular glutamate levels are elevated in the human hippocampus of TLE patients, especially before and during seizure activity [57, 58]. There are at least three ways this can be explained by an astrocyte dysfunction. First, glutamate uptake by astrocytes or its metabolism could be impaired [59]. Second, astrocytic regulation of neuronal excitability could fail leading to increases in neuronal glutamate release. Third, astrocytes could release the additional glutamate themselves.

Regarding astrocytic glutamate uptake, immunostaining studies reported downregulation of the protein levels of both glutamate transporters in human HS [60–62], although other investigators found no changes [63, 64]. The glutamate sensitivity of human glia cells has been assessed in one study by rapid application of glutamate to outside-out patches excised from glia cells in acute hippocampal slices from TLE-HS patients [6]. The results of this study suggest loss of functional transporters and aberrant expression of AMPA receptors in astrocytes, although the identity of the glial cells residing in human HS remained unclear.

It is important to note that glutamate transport is regulated on many levels, which could be important for epileptogenesis. For instance, the mobility of the glutamate transporter GLT-1 on the astrocyte surface has been shown to be activity and location-dependent [65]. Also, glutamate transport is rapidly modulated by burst-like neuronal activity [66], which adds another layer of complexity to glutamate uptake. Importantly, the efficacy of glutamate uptake also depends on the relative position of astrocytic perisynaptic processes and active excitatory synapses. The deletion of the gap junction protein Cx30 for instance resulted in the invasion of the synaptic cleft by astrocytic processes, increased glutamate uptake and decreased excitatory synaptic transmission [67], which links astrocyte gap junction coupling to glutamate homoeostasis. Furthermore, we have recently demonstrated that the relative astrocytic coverage of excitatory synapses correlates with the local efficacy of glutamate uptake and shielding of synapses from invading glutamate from other sources [68], which indicates that epilepsy-associated morphology changes could have a profound effect on glutamate clearance and spread in the tissue. Similarly, perisynaptic astrocytic processes withdraw after the induction of long-term potentiation of synaptic transmission using a high-frequency stimulus, which also increased glutamate spread in the tissue and promoted synaptic crosstalk [69]. This raises the question if transiently increased neuronal activity induces a similar rapid remodeling of astrocytes and whether that promotes or triggers epileptogenesis. The possibility of such a mechanism is also suggested by the following observations. On the one hand, astrocyte morphology is controlled by small GTPases of the Rho family and downstream kinases such as the Rho-associated protein kinase (ROCK) [70, 71]. On the other hand, pharmacological inhibition of ROCK decreased the severity of seizures in the PTZ kindling model [72] and reduced neurodegeneration in a kainic acid epilepsy model [73].

Interesting insights were also obtained about the role of GS in human HS. Here, a pronounced reduction of the enzyme and its functional activity was described [64, 74]. Direct evidence for the involvement of GS deficiency in epilepsy is given by the fact that patients with congenital, homozygous mutations in the GS gene display severe brain malformations and epileptic seizures [75, 76]. Interestingly, experimental induction of reactive gliosis was shown to reduce the expression of GS in the hippocampus, to reduce GABAergic synaptic inhibition but not excitatory synaptic transmission, and to render the hippocampal circuit hyperexcitable [77].

As pointed out above, a failure of astrocytes to limit excitability of neurons could lead to the observed increase of glutamate levels in TLE and HS. Impairment or failure of astrocyte K^+^ buffering is one potential mechanisms (see previous section). Another relevant one is the astrocytic control of the excitability of neuronal networks through extracellular concentrations of adenosine via the enzyme adenosine kinase (ADK). As adenosine exerts powerful anticonvulsive and neuroprotective effects by acting on pre- or postsynaptic A_1_ receptors, alterations in ADK expression are thought to play a crucial role in epilepsy [78, 79]. Baseline adenosine levels in microdialysis samples from epileptic patients are relatively low, while they rapidly rise during seizures, a process hypothesized to mediate seizure termination and postictal suppression [80]. Using immunocytochemistry and Western blot analysis, Aronica and colleagues [81] demonstrated marked overexpression of astrocytic and total ADK protein levels in the sclerotic hippocampi of TLE patients, a phenomenon they considered a common pathologic hallmark of medically intractable chronic epilepsy.

Increased ADK expression and impaired adenosine-mediated inhibition have also been implicated in experimental TLE [78, 82]. For example, knock-down of ADK using ADK-targeting microRNA attenuated kainate-induced acute seizures [83], and pharmacological inhibition of ADK during the chronic phase of KA-induced epilepsy ameliorates seizures [84]. Moreover, overexpression of ADK in the brain induces hyperexcitability and seizures [85, 86], while adenosine augmentation therapies possess seizure suppressing and anti-epileptogenic effects [87–90]. Interestingly, ADK expression depends on the stage of epilepsy, with decreased expression immediately following intrahippocampal kainate injection, but increased expression during the latent or chronic periods (≥ 3 d) of epilepsy [84, 91]. Accordingly, transient administration of an ADK inhibitor during a period of elevated ADK expression in the latent period reduced seizure activity and granule cell dispersion at later stages of the disease [91]. Together these findings support an important role of adenosine in epilepsy and indicate the potential of ADK-targeting and adenosine-enhancing therapies for the treatment of the disease. It should be noted however that ADK is primarily expressed by astrocytes and other glial cells in rodents [92, 93], whereas its expression is more homogeneous across cell types according to human sequencing data [94].

Another possible explanation for the increased glutamate concentrations in epileptic tissue could be excessive astrocytic release of the neurotransmitter. Indeed, astrocytes are believed to not only detect and react to neuronal activity, but also to respond and actively regulate neuronal excitability and synaptic transmission through Ca^2+^-dependent release of neuroactive substances (so-called gliotransmitters like glutamate, ATP, and D-serine) [95, 96]. Such bidirectional signaling between astrocytes and neurons has also been demonstrated in human brain tissue from drug resistant TLE patients, but it remained unclear whether this represented a pathological or a physiological phenomenon, as control tissue was not analyzed [97]. Up-regulation of astrocytic metabotropic glutamate receptors (mGluRs), which are also involved in neuron-glia interactions, has been demonstrated in human TLE [98–101] and may indicate altered gliotransmission. However, direct evidence for an involvement of astrocytic glutamate release in triggering epileptic activity is still missing to our knowledge.

## Dysregulation of Water Flux

Extracellular K^+^ and neurotransmitter concentrations and dynamics are not only dependent on astrocyte uptake and clearance mechanisms, but also on the volume of the extracellular space (ECS), which is regulated by a family of membrane channels termed aquaporins (AQPs). The predominant isoform of aquaporins in adult brain, AQP4, is expressed in close association with Kir4.1 channels in astrocytic perivascular endfeet and perisynaptic processes.

In sclerotic hippocampi from TLE patients the overall expression of AQP4 protein is increased [102, 103], but the density of the water channels along the perivascular membrane domain of astrocytes is reduced. This perivascular AQP4 loss resulted from decreased perivascular expression of the anchoring protein dystrophin and was postulated to perturb the flux of water and K^+^ through astrocytes and consequently increase the occurrence of seizures [102, 103]. As in the case of the Kir4.1 gene, KCNJ10, genetic studies revealed several SNPs in the human AQP4 genes that were associated with TLE [21].

In animal models of epilepsy, AQP4 dysregulation occurs during the early phase of epileptogenesis, which suggest that it is a potential driver of epileptogenesis [104–107]. Moreover, studies employing AQP4 KO mice provided evidence for a causal involvement of AQP4 channels in the disease. For example, AQP4 KO mice are characterized by increased spontaneous recurrent seizures and neuronal loss following kainate-induced SE [107]. Similarly, AQP4 KO mice display increased seizure duration and altered EEG power spectra in experimental posttraumatic epilepsy [108]. Mechanistically, lack of AQP4 may contribute to the pathophysiology of epilepsy due to the role of water channels in regulating ECS volume and osmolarity, as well as its involvement in K^+^ homeostasis [82, 109]. Notably, constitutive deletion of AQP4 is accompanied by enhanced astrocyte gap junction coupling and K^+^ buffering [110], which complicates the interpretation of data from AQP4 deficient mice. Nonetheless, these studies collectively indicate that AQP4 can play an important role in the initiation of epilepsy.

## Conclusions and Future Directions

There is substantial experimental evidence for several astrocytic candidate mechanisms that could initiate the development of TLE. For the sake of conciseness, we have focused on astrocytic K^+^ clearance and glutamate homeostasis and a few relevant aspects, such as K^+^ channels, astrocytic gap junction coupling, glutamate uptake and metabolism, water transport and adenosine signaling (Fig. 1). This list is not exhaustive, but these examples highlight candidate mechanisms supported by particularly strong evidence obtained in epilepsy models of TLE and human TLE.

As discussed above, for a perturbation of astrocyte function (e.g. change of protein expression, function, signaling) to be causal in the development of TLE it needs to meet several requirements. For instance, it must occur before the onset of epilepsy. Also, preventing that astrocytic change should prevent the development of TLE too, for instance in an animal model. Ideally, reproducing that astrocyte change in isolation would also lead to the development of TLE. Establishing causality to this extent remains a challenge. Astrocytic gap junction uncoupling is a good example (see above for details). The uncoupling occurs before the onset of chronic epilepsy. However, complete inhibition of coupling in genetically modified mice does not directly lead to chronic epilepsy, which suggest that more than one factor is involved. Identifying those additional factors remains a challenge. Also, preventing uncoupling experimentally in an epilepsy model is difficult because it requires precise knowledge of how uncoupling was triggered mechanistically. A follow-up question is then if what was learnt about additional factors and mechanisms leading to the development of chronic seizure in a TLE model using, for instance, chemoconvulsants can be transferred to human TLE where the triggers and how they lead to TLE is incompletely understood. Naturally, these considerations apply to any astrocytic candidate mechanism for the initiation of TLE. Using mice where epilepsy-relevant glial candidate genes are selective and inducibly deleted or manipulated might be of great help in answering some of the open questions.

## Abbreviations

ADK: adenosine kinase
AMP: adenosine monophosphate
AQP4: aquaporin 4
BBB: blood-brain barrier
ER: endoplasmic reticulum
GJC: gap junction channel
GS: glutamine synthetase
IP_3_: inositol triphosphate
Kir4.1_3_: inwardly-rectifying K^+^ channel 4.1
mGluR_3_: metabotropic glutamate receptor
PAP_3_: perisynaptic astrocyte process
TGF-ßR_3_: transforming growth-factor ß receptor
